# Coral bleaching under thermal stress: putative involvement of host/symbiont recognition mechanisms

**DOI:** 10.1186/1472-6793-9-14

**Published:** 2009-08-04

**Authors:** Jeremie Vidal-Dupiol, Mehdi Adjeroud, Emmanuel Roger, Laurent Foure, David Duval, Yves Mone, Christine Ferrier-Pages, Eric Tambutte, Sylvie Tambutte, Didier Zoccola, Denis Allemand, Guillaume Mitta

**Affiliations:** 1 UMR 5244, CNRS EPHE UPVD, Université de Perpignan, 52 Avenue Paul Alduy, 66860 Perpignan Cedex, France; 2 Aquarium du Cap d'Agde, 11 rue des 2 freres, 34300 Cap d'Agde, France; 3 Centre Scientifique de Monaco, Avenue Saint Martin, MC-98000 Monaco-Ville, Principality of Monaco

## Abstract

**Background:**

Coral bleaching can be defined as the loss of symbiotic zooxanthellae and/or their photosynthetic pigments from their cnidarian host. This major disturbance of reef ecosystems is principally induced by increases in water temperature. Since the beginning of the 1980s and the onset of global climate change, this phenomenon has been occurring at increasing rates and scales, and with increasing severity. Several studies have been undertaken in the last few years to better understand the cellular and molecular mechanisms of coral bleaching but the jigsaw puzzle is far from being complete, especially concerning the early events leading to symbiosis breakdown. The aim of the present study was to find molecular actors involved early in the mechanism leading to symbiosis collapse.

**Results:**

In our experimental procedure, one set of *Pocillopora damicornis *nubbins was subjected to a gradual increase of water temperature from 28°C to 32°C over 15 days. A second control set kept at constant temperature (28°C). The differentially expressed mRNA between the stressed states (sampled just before the onset of bleaching) and the non stressed states (control) were isolated by Suppression Subtractive Hybridization. Transcription rates of the most interesting genes (considering their putative function) were quantified by Q-RT-PCR, which revealed a significant decrease in transcription of two candidates six days before bleaching. RACE-PCR experiments showed that one of them (*PdC-Lectin*) contained a C-Type-Lectin domain specific for mannose. Immunolocalisation demonstrated that this host gene mediates molecular interactions between the host and the symbionts suggesting a putative role in zooxanthellae acquisition and/or sequestration. The second gene corresponds to a gene putatively involved in calcification processes (*Pdcyst-rich*). Its down-regulation could reflect a trade-off mechanism leading to the arrest of the mineralization process under stress.

**Conclusion:**

Under thermal stress zooxanthellae photosynthesis leads to intense oxidative stress in the two partners. This endogenous stress can lead to the perception of the symbiont as a toxic partner for the host. Consequently, we propose that the bleaching process is due in part to a decrease in zooxanthellae acquisition and/or sequestration. In addition to a new hypothesis in coral bleaching mechanisms, this study provides promising biomarkers for monitoring coral health.

## Background

Coral reefs are fascinating ecosystems, characterized by high levels of biodiversity and ecological complexity, high primary productivity and have significant aesthetic and commercial value, particularly in relation to fisheries, tourism and the aquarium industry. In recent decades coral reefs have been dramatically impacted by large-scale disturbances [1]. Natural disturbances are a routine part of coral reef community dynamics, but they have increased in frequency and severity during the last three decades [1,2]. In addition to providing multiple sources of anthropogenic disturbance that directly kill coral colonies [3], human activities have likely contributed to the increase in natural disturbances via global warming.

Of the broad range of natural and anthropogenic perturbations that affect coral reefs, coral bleaching is recognised as a major disturbance that has the potential to significantly alter the biological and ecological processes that maintain reef communities [4,5]. This phenomenon can occur from the colony scale to the geographical scale where it leads to "mass" coral bleaching with occasional high mortality rates. For example, in 1998 a global mass coral bleaching event led to the death of 16% of the worlds corals [6].

Physiologically, this phenomenon is due to the breakdown of the phototrophic mutualistic symbiosis between scleractinian corals and dinoflagellate endosymbionts (genus *Symbiodinium *spp.), commonly referred to as zooxanthellae. This symbiosis breakdown can be the consequence of a large variety of environmental stressors [7-9], but one of the most important for mass coral bleaching is abnormal high sea surface temperatures which can act synergistically with high solar irradiance [10-12]. At the cellular level, coral bleaching refers to a substantial or partial loss of the endosymbiotic algae from the coral tissues, and/or the loss or reduction of photosynthetic pigment concentrations within zooxanthellae [9].

At the molecular level, the first step of temperature or light induced coral bleaching is the photoinhibition mechanism experienced by the zooxanthellae [13-15]. This often results in the overproduction of reactive oxygen species (ROS) by transport chain electrons [16]. ROS are highly cytotoxic and some of them can easily cross biological membranes leading to severe oxidative stress in both host and symbiotic cells. This oxidative stress can result in the activation of cell necrosis and apoptosis [17,18], which represent two of the six identified ways of endosymbiotic loss during bleaching [18-23]. The four other ways of symbiont disappearance are: i) *in situ *digestion of zooxanthellae by the coral host [24,25], ii) expulsion by exocytosis or iii) by pinching off [24,26], and iv) host cell detachment [22].

The increase in the incidence and magnitude of coral bleaching episodes in recent decades [27] and the present context of global warming [5] strengthens the interest in this research field. Studies on early molecular mechanisms triggering and leading to these different ways of symbiotic loss are necessary to better understand the phenomenon and can provide early molecular markers to monitor coral health. In this context, several comparative molecular studies were undertaken. They compared healthy, semi-bleached and bleached corals or symbiotic *versus *aposymbiotic anemones. These studies revealed different genes involved in the response to the stress as well as genes putatively involved in bleaching mechanism or symbiosis breakdown/onset [28-35]. Some of the genes identified in these different studies are promising candidates to explain bleaching processes but studies on their expression and function are now necessary to validate their putative role. For example, a recent study of Desalvo et al. (2008) evidenced a putative calcium homeostasis disruption that could trigger different cellular processes leading to cell death via apoptosis and necrosis. Concerning symbiosis regulation, *Sym 32*, a fasciclin domain containing protein, was shown to be differentially expressed between symbiotic and aposymbiotic anemones, could be involved in host/symbiont interaction and symbiosis breakdown under cadmium exposure [34,36,37].

In the present study, the experiment was specifically designed to identify genes regulated in the early stages of the thermal stress process leading to bleaching. To achieve this aim, we developed a comparative transcriptomic approach (by Suppression Subtractive Hybridization) comparing stressed corals before bleaching symptoms appears *versus *controls. Because our aim was also to develop functional markers that could be used to monitor coral health, we choose *Pocillopora damicornis *as a model species due to its widespread distribution in the Indo-Pacific region [38] and its high sensitivity during mass bleaching events [39-42]. Our SSH approach led to the identification of two genes displaying an important down-regulation just before bleaching. Characterization of their precursors and immunolocalization experiments revealed their putative function and permitted the emergence of new hypotheses on coral bleaching mechanisms. In addition, these two genes constitute promising biomarkers for coral health monitoring.

## Results

### Bleaching monitoring

**Figure 1:**
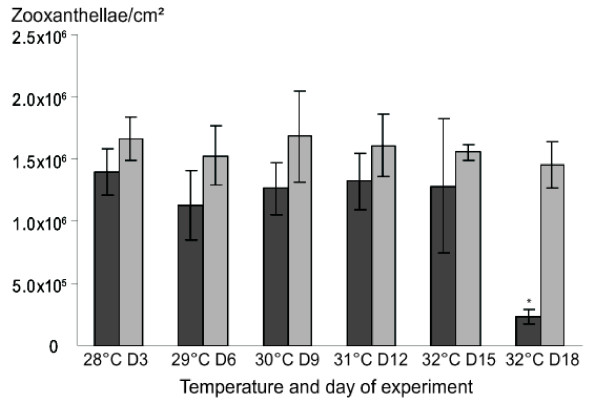
**Zooxanthellae density measured during the temperature stress**. Dark grey histograms refer to the stressed set, light grey histograms to the control set. The Kruskal-Wallis H-test shows significant differences in the stressed set (P < 0.05). The significant differences (P < 0.05) were identified using the Mann-Whitney U-test and indicated by a star.

Zooxanthellae density and statistical tests (Figure 1) showed that bleaching occurred in the stressed set of corals at 32°C, 15 days after the beginning of the protocol and was complete by the 18^th ^day. In the control set, no bleaching or paling was observed throughout the experiment. After 15 days at 32°C, bleaching induced a large increase in the standard deviation (SD) of zooxanthellae density in comparison to the other points of the kinetic, suggesting that symbiosis in some corals was starting to break. Bleaching was well-established 18 days after the beginning of the protocol with a 79.9% reduction in zooxanthellae density.

In order to select genes regulated early during bleaching, we performed the SSH with samples taken at day 12 and 15. This choice was also driven by an experimental constraint whereby stressed and control samples must have similar zooxanthellae densities to avoid false positive transcripts due to a differential representation of zooxanthellae between the two conditions.

### EST sequencing, general characteristics of the BIG and BRG libraries, gene validation and selection

Two SSH experiments were performed resulting in the construction of four cDNA libraries. The first SSH experiment was the result of the subtractive hybridization between the 31°C (D12) and control sets, and the second experiment between the 32°C (D15) and control sets. One hundred clones from each library were sequenced. To simplify the analysis, putative induced clones and putative repressed clones of the SSH libraries were respectively pooled in two new libraries called "Bleaching Induced Genes" (BIG) and "Bleaching Repressed Genes" (BRG). After sequencing, 147 and 118 high quality cDNA sequences were obtained from the BIG and the BRG libraries, respectively. BIG library ESTs coalesced into 15 contigs and 96 singletons, suggesting that the overall redundancy of the library was about 10.2% (Table 1). The BRG library coalesced into 17 contigs and 70 singletons for an overall redundancy of 14.4% (Table 1). In order to minimize redundancy in the EST database, sequences displaying 100% identity were submitted as a single sequence. ESTs aligning in the same contig but displaying differences in their nucleotidic sequence were submitted individually to the database. A total of 61 and 94 ESTs were submitted to the dbEST section of the NCBI/GenBank database for the BIG and BRG libraries respectively (GenBank: GH706795 to GH706855 and GH706856 to GH706949, for BIG and BRG library respectively). ESTs were subjected to BLASTN and BLASTX searches. Sequence similarities were considered to be significant when the expected value (e value) was less than 10^-2^. Gene encoding proteins involved in photosynthesis, oxidative detoxification, intracellular signalling pathway, metabolism, cytoskeleton structure, conserved protein domains, calcium homeostasis, cell/cell or cell/ligand interactions, protein degradation, chaperone protein and protein synthesis were selected for further analysis [see additional file 1 for, Top blast, GeneBank accession number and specific primers used for Q-RT-PCR]. Among the tested clusters (data not shown), only clusters 12 and 27 showed a drastic regulation. They display 101 and 10.1 fold transcript decreases under stress, respectively.

**Table 1:** General characteristics of the BIG and BRG libraries.

	**BIG library**	**BRG library**
**Sequenced clone**	200	200

**Analysed cDNA**	147	118

**Average insert size (bp)**	360	429

**Average EST size (bp)**	310	308

**EST contigs**	15	17

**Singletons**	96	70

Redundancy* (%)	10.2	14.4

* R = nEST assembled in cluster/Total EST

Clusters 12 and 27 belong to the functional class of cell/cell or cell/ligand interaction and their putative functions make them promising candidates as key factors of symbiosis breakdown/maintenance. Consequently, we decided to focus the remainder of the present study on these genes.

### Cluster 12 and 27 ORF characterization and protein structure

RACE-PCR experiments were performed to obtain the complete ORF of the two selected genes. The cDNA corresponding to cluster 12 displayed significant similarities (E value = 3.10^-7^) with a predicted protein of *Nematostella vectensis*. The precursor displays a cysteine array (InterProScan) shared by snake toxins and some proteins involved in cell adhesion (the uPAR/Ly6/CD59/Snake toxin domain super-family). This gene was named *Pdcyst-rich*, for *Pocillopora damicornis *cystein-rich. The second gene (cluster 27) displayed significant similarities (E value = 1.10^-27^) for the Millectin protein, isolated from *Acropora millepora *[43]. Domain analysis using the InterProScan software showed that this protein contained a DC-SIGN domain characteristic for lectins of the C-type. Consequently, this gene was named *PdC-Lectin*.

cDNA corresponding to *Pdcyst-rich *(GenBank: FJ628421) displays an ORF of 441 base pairs corresponding to a precursor of 147 amino acids (AA). The analysis of the primary structure by PSORTII prediction software reveals that it has a secretory protein-like structure with a 23 peptide signal sequence. Between the residue 25 and 133 of the precursor a domain similar to the uPAR/Ly6/CD59/Snake toxin family was identified using the InterProScan software. The size range of this domain is comprised between 70 and 92 AA [44]. This type of domain is present in a large variety of proteins involved in different functions, including T-lymphocyte activation (Ly-6, [45,46]), fibrinogen formation (uPAR, [47,48]), inhibition of the complement mediating lysis (CD59, [44]) and snake venom [49]. The principal structural features of this domain are the presence of (i) 8 to 10 cysteine residues involved in di-sulphide bond formation, and (ii) a typical motif "CCXXDXCN" at the C-terminal end of the domain [50]. As the proteins sharing the uPAR/Ly6/CD59/Snake toxin domain are classically N-glycosylated and GPI anchored, the N-glycosylation and GPI anchored status of the *Pdcyst-rich *protein were investigated using NetGlyc and PSORT2 software [51,52]. The N-glycosilation site was predicted on the 75^th ^residue of the precursor and a GPI anchored signal was found at the C-Terminal end of the protein (residues 144-147).

**Figure 2:**
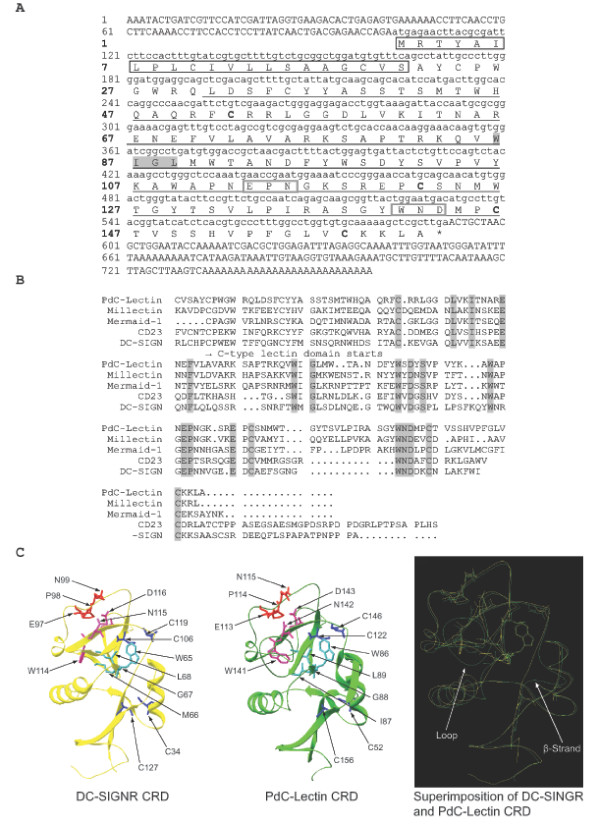
**Sequence and structure analysis of PdC-Lectin gene and protein**. A) cDNA and derived amino acid sequence of *PdC-Lectin*. Black boxes indicate a predicted signal peptide. Underlined area indicates the sequence span of an identified C-type lectin domain. Grey boxes denote conserved mannose binding motifs in a Ca^2+^-dependent manner. Cysteines belonging to the C-type lectin conserved array are in bold. The WIGL motif is highlighted in grey. B) the alignment of C-type lectin domains of PdC-lectin with different C-type lectin domains contained in several similar proteins identified by Blast searches. Conserved amino acid positions are highlighted. Accession numbers: Millectin (GenBank: EU717895), human CD23 (GenBank: P06734), human DC-SIGN (GenBank: Q9NNX6), Mermaid-1 (GenBank: AY927371). C) the 3D structure of PdC-Lectin CRD. Crystal structure of DC-SIGNR CRD (yellow) and molecular model of PdC-Lectin CRD (green). Superposition of the crystal structure of DC-SIGNR CRD and molecular model of PdC-Lectin CRD (C). All conserved motifs are highlighted; "WIGL" is in clear blue, "EPN" in green, "WND" in pink. The four highly conserved cysteines are in dark blue.

cDNA corresponding to *PdC-Lectin *(GenBank: FJ628422) displayed an ORF of 486 base pairs corresponding to a precursor of 162 amino acids (AA; Figure 2A). The analysis of the primary structure by PSORTII prediction software revealed that it has a secretory protein-like structure with a 22 AA peptide signal sequence. The following 140 AA corresponded to a C-type lectin-like domain (CTLD) shared by a large group of extracellular Metazoan proteins [53]. This domain is involved in recognition and binding of carbohydrates in a Ca^2+^-dependent manner [54]. The alignment of *PdC-Lectin *with the most similar CTLD (Millectin of *Acropora millepora*, Mermaid-1 of *Laxus oneistus*, CD 23 and DC-SIGN from *Homo sapiens*), showed the presence of highly conserved cysteine residues (Position: 52, 122, 146, and 158 on the precursor; Figure 2B). These residues are involved in the three dimensional structure by di-sulphide bond formation. C1 (position 52) and C4 (158) link β5 and α1 (the whole domain loop), and C2 (122) and C3 (146) link β3 and β5 (the long loop region, involved in Ca^2+^-carbohydrate binding and domain-swapping dimerization) [53]. The "WIGL" motif present between the residues 63 and 68 is involved in the formation of all tree hydrophobic cores and contributes to structure stabilisation [53]. The presence of all conserved motif and key residues underlines the hypothesis that *PdC-Lectin *CTLD is functional [53]. The highly conserved motifs "EPN" and "WND" in the CTLD (Figure 2A) argue in favour of the specificity for mannose binding in a Ca^2+^-dependent manner [53]. In addition, the MODWEB server [55] was used to perform the homology modelling of PdC-Lectin. It provides a molecular model based on the crystal structure of the human DC-SIGNR carbohydrate recognition domain (CRD) [56] and presented a model-score of 1.00 (a model is predicted to be good when the model score is higher than 0.7). The molecular model (Figure 2C) revealed that all residues involved in the binding of mannose in a Ca^2+^-dependent manner (EPN and WND) are located in similar structures (a long loop region for EPN and a β strand for WND; Figure 2C). Moreover, the four conserved cysteines and the "WIGL" motif known to be involved in the maintenance of the CTLD fold have a conserved position in the 3D structure (see superimposition of Figure 2C). The differences (one loop and one β-strand) which can be observed between the template and the molecular model are located in regions of the molecule that are not considered as essential for function (see Figure 2C).

### Expression rates of *PdC-Lectin *and *Pdcyst-rich *in comparison to classical indicators of bleaching

**Figure 3:**
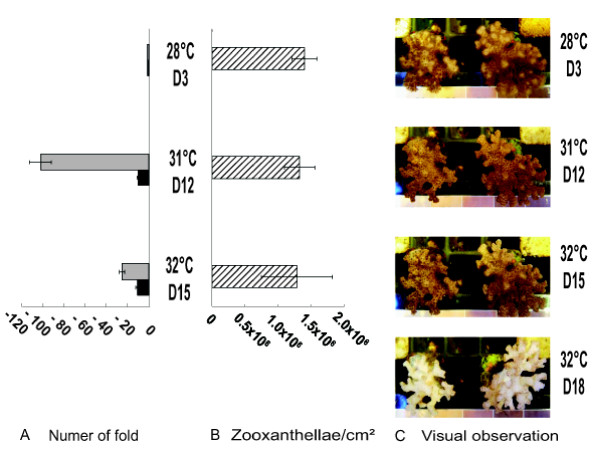
**Transcription rates of *PdC-Lectin *and *Pdcyst-rich *in parallel with the classical indicator of bleaching**. Expression rate (A) of *PdC-Lectin *(Black histogram) and *Pdcyst-rich *(Grey histogram) and the corresponding zooxanthellae density (B, Hatched histogram) at 28°C (day 3, D3), 31°C (D12) and 32°C (D15). Pictures of coral nubbins at the corresponding stages are shown (C).

Q-RT-PCR were performed on RNA extracted from coral nubbins sampled at 28°C (D3), 31°C (D12) and 32°C (D15) of the kinetic to follow the transcript variations corresponding to *PdC-Lectin *and *Pdcyst-rich *during the stress protocol (Figure 3). Transcript amounts were expressed as a relative ratio to the control values obtained at 28°C (D3). For both genes, although zooxanthellae densities were stable until day 15 (Figure 3B), transcript decreases were observed after 12 days (Figure 3A). Indeed, 101 and 10.1 fold decreases were measured on the 12^th ^day (D12) of the protocol for *Pdcyst-rich *and *PdC-Lectin *genes. At 32°C (D15), the zooxanthellae decrease was non-significant and the levels of *Pdcyst-rich *and *PdC-Lectin *transcripts remained low (25 and 11 fold decreases, respectively compared to control, Figure 3B).

In conclusion, these two genes were drastically down regulated at least six days before the first usual symptoms of bleaching (visual observation, Figure 3C and zooxanthellae density decreases, Figure 3B).

### Genes corresponding to *PdC-Lectin *and *Pdcyst-rich *are expressed by coral cells

**Figure 4:**
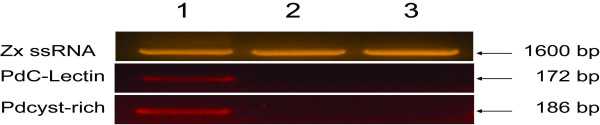
***PdC-Lectin *and *Pdcyst-rich *are expressed by the host**. Presence of *PdC-Lectin *and *Pdcyst-rich *genes and the zooxanthellae small ribosomal subunit RNA (Zx ssRNA) corresponding gene are investigated by PCR using specific primers on DNA extracted from holobionts (corals plus zooxanthellae, lane 1) or zooxanthellae isolated from *S. pistillata *and *G. fascicularis *(lanes 2 and 3, respectively).

In order to determine the organism (host or symbiont) expressing each candidate gene we developed cross PCR experiments performed on DNA extracted from the holobiont (host plus symbiont, Figure 4, lane 1) and from pure cultured *Symbiodinium *spp. (isolated from *S. pistillata *and *G. fascicularis*, Figure 4, lane 2 and 3, respectively). These PCR were performed with oligonucleotides specifically amplifying both genes of interest and *Symbiodinium *spp. specific primers (ss5Z and ss3Z) identified in a previous study [57] and known to amplify small ribosomal subunit RNA. Whereas primers specific to genes encoding *PdC-Lectin *and *Pdcyst-rich *proteins only amplified DNA extracted from holobionts (Figure 4, lane 1), ss5Z and ss3Z primers amplified all DNA tested (Figure 4, lanes 1, 2 and 3). This last result demonstrated that PdC-Lectin and Pdcyst-rich protein corresponding genes are specific for corals and confirmed similarity results obtained after database comparisons (see above).

### Immunolocalization of PdC-Lectin and Pdcyst-rich proteins

In order to further examine the location of PdC-Lectin and Pdcyst-rich proteins within coral tissues, antibodies were raised against synthetic peptides designed from PdC-Lectin and Pdcyst-rich primary structures. Initially, the specificity of the antibodies was tested by Western blot experiments on holobiont extracts which revealed a single band in both cases (data not shown).

**Figure 5:**
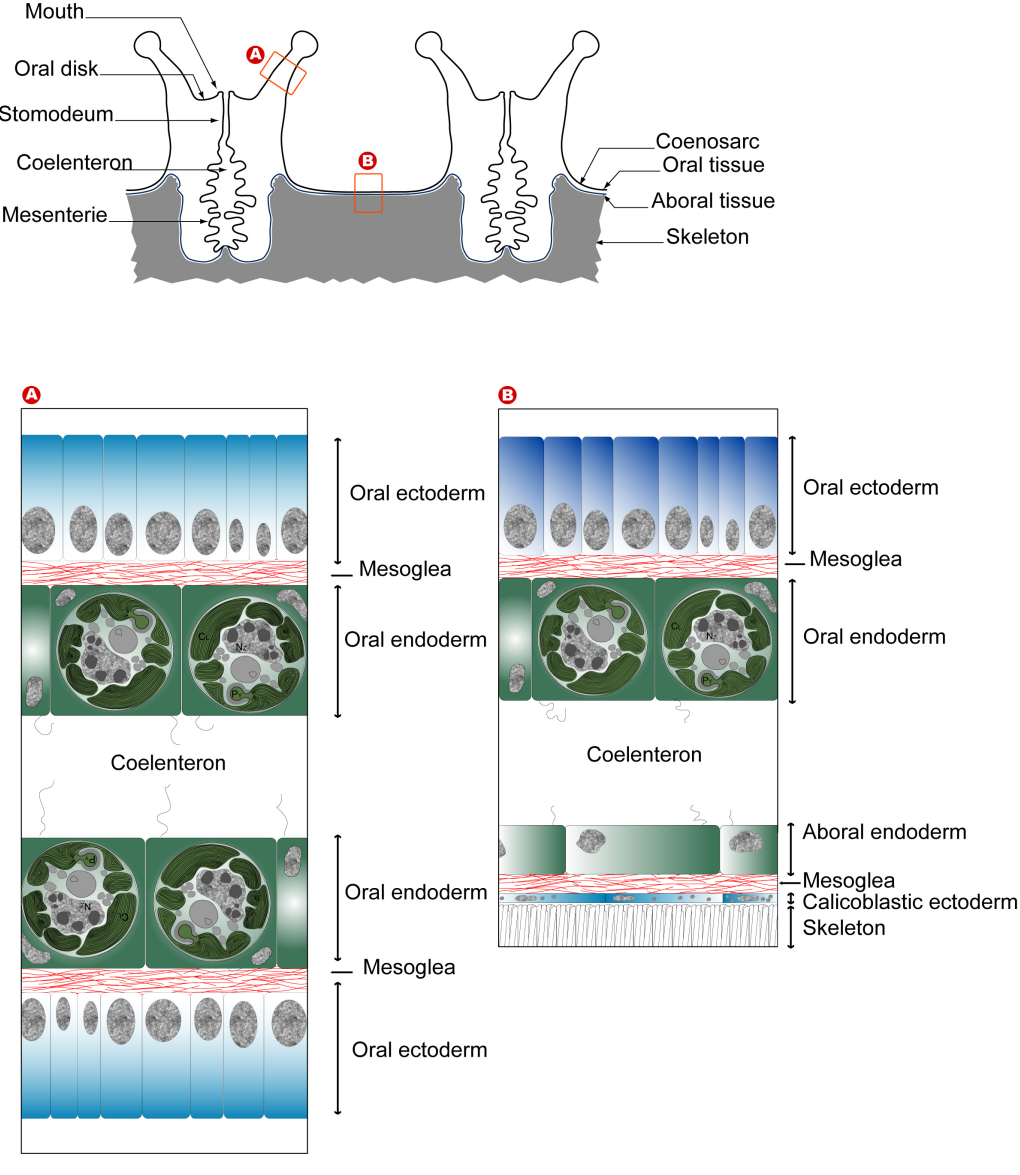
**Schematic representation of the anatomy and histology of *P. damicornis***. (A) the histology of the tentacle composed of oral tissues and (B) the histology of the coenosarcs composed of oral and aboral tissue.

In order to help the reader interpret immunolabeling observations, a schematic representation of the anatomy and histology of *P. damicornis *is provided in Figure 5. *P. damicornis *is a colonial coral characterized by the presence of numerous polyps, linked together by a common tissue usually referred to as the coenosarc (Figure 5). In the polyps, the tentacles are only composed of oral tissue (Figure 5A) whereas the coenosarc is composed of oral and the aboral tissues (Figure 5B). Each of these tissues is composed of an ectoderm separated from the endoderm by an acellular layer of mesoglea (Figure 5B). The oral endoderm faces the coelenteron (gastric cavity) and contains intracellular symbionts commonly called zooxanthellae. The aboral ectoderm faces the skeleton, is composed of calicoblastic cells and is referred to as the calicoblastic ectoderm or calicodermis.

**Figure 6:**
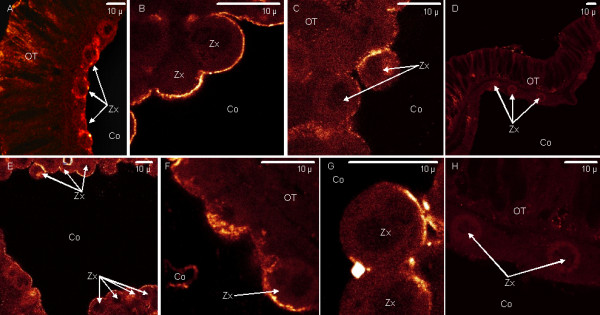
**PdC-Lectin immunolabeling in endodermal cells of the oral tissue in tentacles**. Anti-PdC-Lectin coupled to Alexafluor 568 is revealed in orange (A), (B), (C), (E), (F) and (G). (D) and (H) represent control experiments performed with anti-PdC-Lectin antibodies pre-adsorbed with the synthetic peptide used for immunization. (A), (D) and (E) are large views of the oral tissue (OT) facing the coelenteron (Co) of coral. (B), (C), (F), (G) and (H) correspond to magnifications of coral cells in contact with the coelenteron (Gastric cavity). Other abbreviations: Zx zooxanthellae.

**Figure 7:**
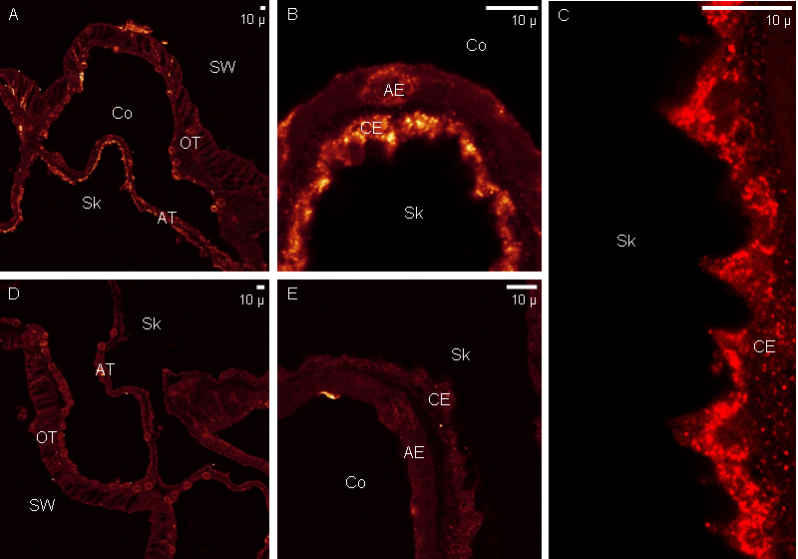
**Pdcyst-rich protein immunolabeling in calicoblastic cells**. Anti-Pdcyst-rich protein coupled to Alexafluor 568 is revealed in orange (A), (B) and (C). (D) and (E) represents control experiments performed with anti-Pdcyst-rich protein antibodies pre-adsorbed with the synthetic peptide used for immunization. (A) and (D) are large views of oral tissue (OT) and aboral tissues (AT) of the coenosarc. (B), (C) and (E) correspond to magnifications of the aboral tissue showing the aboral endoderm (AE), and the calicoblastic ectoderm (CE). Other abbreviations: Co coelenteron, Sk skeleton, SW seawater.

Immunolabeling with anti-PdC-Lectin and anti-Pdcyst-rich protein antibodies is shown in Figure 6 (tentacles) and Figure 7 (coenosarc). Distinct tissues were immunolabeled: anti-PdC-Lectin antibodies labeled the oral endoderm containing intracellular zooxanthellae (Figure 6A) whereas anti-Pdcyst-rich antibody labeled the aboral ectoderm (Figure 7A). When tissues were observed at higher magnification, the immunolabeling with anti- PdC-Lectin antibodies showed a peripheral pattern adjacent to or in the cellular membrane of the endoderm in contact with the coelenteron (Figure 6B, E and 6F). Immunolabeling appeared clearly associated with the membranes (Figure 6B) and to granular structures located next to the membranes (Figure 6F). Additionally, in some cases the labeling was observed at the interface between free zooxanthellae and endodermal host coral cells (Figure 6C and 6G).

Anti-Pdcyst-rich antibodies labeled the calicoblastic ectoderm (Figure 7A) which faced the skeleton in calcified samples. At higher magnifications, it appeared that the immunolabeling of calicoblastic cells was granular and intracellular (Figure 7B, C).

The specificity of the immunolabeling was checked by pre-adsorption of anti-PdC-Lectin and anti-Pdcyst-rich protein IgG with the corresponding synthetic peptides used for immunizations. After this treatment, sections of tissues were no longer labeled (Figure 6D, H and Figure 7D, E) demonstrating the specificity of the immunolabeling.

## Discussion

The aim of the present study was to identify molecular actors involved in the breakdown of the phototrophic mutualistic symbiosis between a scleractinian coral (*Pocillopora damicornis*) and its dinoflagellate endosymbiont (genus *Symbiodinium *spp.) during thermal stress. In contrast to some previous studies, thermal stress conditions used in the present work corresponded to natural conditions in terms of amplitude and rapidity of temperature increase. After SSH and validation steps, two genes belonging to the functional classes of cell/cell or cell/ligand interaction present an important down-regulation. Their down-regulation and putative function make them very promising candidates as key indicators of symbiosis breakdown/onset. Consequently, we decided to focus the remainder of the present study on these genes.

The first gene, *Pdcyst-rich*, displayed significant similarities with a predicted protein of *Nematostella vectensis*. Their complete ORF characterization revealed that its precursor displays a putative signal of secretion and all key features shared by the uPAR/Ly6/CD59/Snake toxin family. As this domain is common to proteins involved in a large variety of functions, structural elements are not sufficient to provide hypotheses about the function of *Pdcyst-rich*. PCR experiments revealed that the corresponding gene is expressed in coral cells and complementary immunolocalization experiments showed that the protein displays a granular location in the calicoblastic ectoderm in contact with the skeleton. As *Pdcyst-rich *proteins are synthesized and stored in granules of this skeletogenic tissue, we investigated the suspected role of proteins of the uPAR/Ly6/CD59/Snake toxin family in mineralization process. Two proteins were interesting in this context: RoBo-1 [58] and HEP21 [59]. The function of these proteins remains elusive but their involvement in mineralization process of bones and eggs was hypothesized [58,59]. All these data taken together and in particular the granular location of Pdcyst-rich proteins in calicoblastic cells, suggests that Pdcyst-rich proteins could also play a role in the mineralization process. As it was shown that stress has an immediate effect triggering growth and calcification arrest in scleractinian corals [10,60-62], the strong decrease of the transcript corresponding to Pdcyst-rich protein could reflect the trade-off mechanism occurring during stress and leading to the arrest of the mineralization process. A recent transcriptomic study analyzing differential gene expression during thermal stress in *M. faveolata *also provides evidence of transcript decreases of genes involved in calcification [29].

The second gene identified in the present study was named *PdC-Lectin*. It contains a putative signal peptide and a C-type lectin-like domain shared by a large group of extracellular Metazoan proteins with diverse functions [53]. This domain is involved in recognition and binding of carbohydrates in a Ca^2+^-dependent manner [54]. Alignments and diverse structural analyses revealed that PdC-Lectin is functional and shares the mannose binding specificity characteristics of the human DC-SIGN CRD. The presence of the highly conserved motif "EPN" and "WND" in the CTLD of PdC-Lectin argues in favour of the specificity for mannose binding in a Ca^2+^-dependent manner [53] and the molecular model obtained using the MODWEB server reveals that all residues involved in this binding specificity are located in similar predicted structures by comparison with the human DC-SIGNR CRD. Moreover, the differences observed between the template (human DC-SIGNR CRD) and the PdC-Lectin molecular model are located in regions of the molecule that are not considered as essential for function. In addition, PdC-Lectin displays high similarities (E value = 1.10^-27 ^and see alignment Figure 2B) for lectins recently characterized from *Acropora millepora *[43]. These lectins, named Millectin, were isolated by mannose affinity chromatography and were shown to bind to bacterial pathogens as well as coral symbionts, dinoflagellates of the genus *Symbiodinium*. All these elements taken together strengthen the hypothesis that PdC-Lectin is functional and shares the mannose binding specificity of the human DC-SIGN CRD.

Recent work has highlighted the key role played by lectin/glycan interactions in symbiosis onset [63,64]. The Virginia Weis group provided evidence for a recognition mechanism involving lectin/glycan at the onset of symbiosis between aposymbiotic larvae of the coral *Fungia scutaria *and their endosymbiotic zooxanthellae [64]. They showed that algal cell surface glycan ligands, such as α-mannose/α-glucose and α-galactose play a role in recognition during initial contact at the onset of symbiosis [64]. A second study described the role of another lectin, called Mermaid-1 [63]. This protein displayed the same structural features as PdC-Lectin sharing the same DC-SIGN domain. Mermaid-1 mediates symbiotic bacteria acquisition by the marine nematode *Laxus oneistus*. In this thiotrophic symbiosis, a monolayer of symbiotic sulphur-oxidizing bacteria covers the cuticle of the nematode. The authors showed that this secreted Ca^2+^-dependent mannose-specific lectin is capable of inducing symbiont aggregation and acquisition [63].

The different structural and bibliographic elements described here argue in favour of a putative role for PdC-Lectin in recognition and binding between the host and the symbionts. The putative signal of secretion, the structural similarities with these different proteins sharing the DC-SIGN domain and its mannose binding specificity suggest that PdC-Lectin could interact with zooxanthellae mannose ligands to mediate symbiont acquisition as it was described for the *F. scutaria*/*Symbiodinium *model [64]. In order to strengthen this hypothesis we performed expression analysis experiments. We confirmed the expression of *PdC-Lectin *in coral cells but the most interesting finding was obtained using immunolocalization experiments where we found evidence of PdC-Lectin immunoreactivity in coral cells belonging to endodermal tissue. This labeled tissue is in contact with free zooxanthellae that are transitorily present in the coelenteron (gastric cavity). We observed a peripheral immunostaining pattern adjacent to or in the cellular membrane in contact with the coelenteron. In addition, some free zooxanthellae were observed in contact with endodermal cells and interestingly, as shown in Figure 6C and 6G, the labeling appears at the interface between zooxanthellae and host coral cells. These data strongly suggest a putative role of PdC-Lectin in zooxanthellae interaction and acquisition.

Finally, our hypothesis is strengthened by the diminution of the *PdC-Lectin *transcript level during stress and during symbiosis breakdown. *PdC-Lectin *transcript concentration decreases just before bleaching occurs and remains low during bleaching process. This means that this gene could be regulated just before symbiosis breakdown. Consequently, and in agreement with the previous data obtained, we propose that bleaching is due in part to a decrease in zooxanthellae acquisition and/or sequestration.

## Conclusion

This novel hypothetical mechanism could appear in conjunction with different cellular events reviewed in a recent paper [65]. In this review, the author examined the cellular events leading to the collapse of symbiosis during heat and light stress. Briefly, ROS is shown to play a central role in both injuries to the partners and to inter-partner communication of a stress response. This review also presented evidence that bleaching is a host innate immune response against symbionts. Finally, the different ways of elimination or removal of the symbiont tissues are described through a variety of mechanisms including exocytosis, host cell detachment and host cell apoptosis. Our work reveals another interesting hypothesis with the inhibition of zooxanthellae acquisition processes. During heat stress zooxanthellae could be considered as toxic due to the over-production of ROS and therefore eliminated by host immune responses.

Future work focusing on *PdC-Lectin *function has to be conducted to verify our hypothesis. We could study the mechanisms of zooxanthellae acquisition during the resilience process following experimental bleaching. Recombinant PdC-Lectin and antibodies directed against recombinant PdC-Lectin could be used to facilitate or inhibit the acquisition process of cultured and labeled zooxanthellae during resilience. This functional validation is necessary to be sure of having an early marker of thermal bleaching.

This marker could be used by coral reef managers to (i) distinguish between different stresses on corals, and (ii) precisely and accurately predict bleaching events in conjunction with temperature anomalies indices such as "Degree Heating Weeks" or "Hotspot" developed by the NOAA. This will help managers in the implementation of policy responses and compensatory measures. In addition, these functional biomarkers of thermal stress may be used to evaluate the health of coral transplants kept in public aquaria, in coral farms and for coral nubbins transplanted during restoration projects as these entities are key factors in coral reef conservation in a sustainable development and global warming context.

## Methods

### Biological material

The *Pocillopora damicornis *isolate used in the present study was harvested in Lombock (Indonesia, CITES number: 06832/VI/SATS/LN/2001) and maintained at the Cap d'Agde Aquarium (France). For our experimental stress, *P. damicornis *nubbins (10 g; 7 cm high; 6 cm in diameter) were propagated by cutting branches from parents and then fixing on a concrete support. To avoid toxicity and to facilitate fixation of the nubbins, the concrete supports were first rinsed and biologically stabilized by immersion in aquaria for two months. The nubbins were used after one month to allow complete cicatrisation and physiological stabilization [66]. During this cicatrisation step, nubbins were maintained at 27°C.

In order to determine which cells (host or symbiont) were expressing the candidate genes, two zooxanthellae isolates were used. The first corresponds to clonal cultures of zooxanthellae (clade A [67]) originally isolated from a Red Sea colony of *Stylophora pistillata *[68]. The second corresponds to clonal cultures of zooxanthellae (clade B) originally isolated from a Red Sea colony of *Galaxea fascicularis *[69]. Both zooxanthellae clades were cultured in 250 mL screw-top polycarbonate Erlenmeyer flasks (Corning^®^) in modified ASP-8A medium [70] at pH 8.2. The zooxanthellae were grown in an incubator at 26 ± 1°C under a PAR irradiance of 150 μmol photon.m^-2^.s^-1 ^(≈ 33 W m^-2^) from Sylvania Gro Lux^® ^and daylight fluorescent tubes, on a 12:12 h (light:dark) photoperiod. The stock cultures were transferred monthly. Cells were used in stationary phase.

### Thermal stress protocol

**Figure 8:**
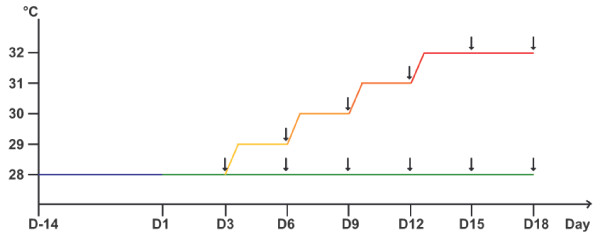
**Schematic representation of the experimental design**. After a 14 days-acclimatization step at 28°C (D-14 to D1, blue line), the stressed set (yellow to red line) was submitted to a gradual water temperature increase of one degree every 3 days until 32°C was reached (D3, D6, D9, D12, and D15). This last temperature was maintained until corals bleached (D18). In parallel, the control set (green line) was maintained at 28°C all along the experiment. Sampling times are indicated by arrows. Three nubbins were sampled for each time of the kinetic and each treatment.

Forty-six nubbins were randomly distributed between two sets (one stressed set and one control set) contained in two 200 L tanks (110 cm × 90 cm × 20 cm). These colonies were then acclimatized to a temperature of 28°C for two weeks [62]. This temperature corresponds to the sea surface temperature observed during the warmer months in most coral reefs around the world [11,60,71-76]. To create stress conditions triggering an *in situ *mass bleaching event, the temperature of the stressed set was increased by about 1°C every three days, until 32°C was achieved (D15). This last temperature corresponds to the maximal temperature observed in different coral reefs during mass bleaching events [11,60,71-76]. This temperature threshold was then maintained until bleaching occurred (D18) while the control set was maintained at 28°C (Figure 8). For each condition (stressed and control), three nubbins were randomly sampled at the end of each step (every three days). Three apexes of 0.5 cm were cut from each nubbin for zooxanthellae density measurements. Nubbins and apexes were frozen and stored in liquid nitrogen until analysed.

Aquaria temperatures were controlled by connecting an aquarium heater (600 W, Schego) to an electronic thermostat (Hobby Biotherm Professional). Aquaria were illuminated by metal halide lamps (Osram Day Light 5200 K, 400 W) providing an irradiance of 350 μmol photon.m^-2^.s^-1 ^(quantum meter: QMSW-SS; by Apogee Instruments Inc.) on a 12:12 h (light:dark) photoperiod. All other seawater characteristics (e.g., salinity 36 g.L^-1^, pH 8.3) were maintained constant and equal for each set. A constant water flow was maintained in each tank using a water pump (Tunze Nanostream 6045, delivery 4500 L.h^-1^). Seawater was continuously recycled at rate of 12 tank volumes per hour by coupling the action of a biological filter and an Aquavie protein skimmer (model: PS 1200), and refreshed by natural filtered Mediterranean seawater heated to 28°C at a renewal rate of 2.2 tank volumes per hour.

### Bleaching monitoring

Zooxanthellae density was monitored during the whole experiment in order to follow the bleaching process. Coral tissues were extracted using a Water Pick [77] in 0.5 μ of filtered sea water. The slurry was homogenised using a Potter grinder and zooxanthellae were counted using an improved version of the Histolab 5.2.3 image analysis software (Microvision, Evry, France) [78]. Zooxanthellae number was then standardized per skeletal surface area, using the wax protocol described in [79].

The normality of the data set was assessed using the Kolmogorov-Smirnov test. As data was not normally distributed we used non-parametric statistical procedures. The Kruskal-Wallis H-test was used to compare the zooxanthellae density measures within each set. The Mann-Whitney U-test was used to compare the zooxanthellae density measures at each point on the kinetic between the stressed and control sets. All statistical analyses were conducted using GraphPad instat 3 (Kruskal-Wallis) and SPSS 10.0 (Kolmogorov-Smirnov and Man-Whitney). α was set at 0.05 for all analyses.

### RNA extraction and mRNA purification

Tissues from three nubbins (sampled for each stress and control conditions) were harvested using a Water Pick in 800 ml of 0.5 μ filtered seawater refrigerated at 4°C. Extracts were pooled and centrifuged at 2000 g for 10 min at 4°C. Supernatant was discarded and pellets were homogenized in 15 ml of Trizol (Invitrogen). Total RNA was extracted following manufacturer instructions. mRNAs were purified using the NucleoTrap mRNA mini kit (Macherey-Nagel).

### Subtractive cDNA library construction

Forward and reverse libraries were constructed by subtracting mRNA from stressed nubbins with mRNA from control nubbins. Two independent experiments were performed: D12 stressed *versus *control corals and D15 stressed *versus *control corals. SSH libraries were produced using the PCR-Select cDNA subtraction kit (Clontech). Tester and Driver cDNA were prepared using 2 μg of poly(A)^+ ^RNA. Enzyme digestion, adapter ligation, hybridization, and PCR amplification were performed according to protocols provided by the manufacturer (Clontech). PCR products were cloned into pCR4-TOPO cloning vectors using the TOPO TA cloning kit (Invitrogen) and transformed into One Shot TOP10 chemically competent *Escherichia coli *cells (Invitrogen).

### DNA Sequencing, sequence analysis and modelling

For each library, 100 clones were randomly selected and single pass sequenced using a dideoxy-dye-terminator method (CEQ™ DTCS-Quick Start Kit, Beckman coulter) and a CEQ™ 8000 apparatus (Beckman Coulter). Vector and adaptor sequences were trimmed from all sequences using Sequencher™ software (Gene Codes Corporation). High quality ESTs, longer than 150 bp in length, were assembled in clusters or unique sequences from singletons and submitted to database searches using BLASTX and BLASTN programs [80]. Specific domain searches were performed using InterProScan program [81]. EST sequences have been submitted to the dbEST database.

Homology modelling was performed on MODWEB [55] and the protein diagrams constructed on Swiss-PdbViewer 4.0.1 [82]. The three-dimensional (3D) structure comparison between the template and the molecular model was made using Superpose web server [83].

### Real Time PCR

Gene encoding proteins involved in photosynthesis, oxidative detoxification, intracellular signalling pathway, metabolism, cytoskeleton structure, conserved protein domains, calcium homeostasis, cell/cell or cell/ligand interactions, protein degradation, chaperone protein and protein synthesis were selected for quantitative analysis of their transcripts. Real-Time RT-PCR was performed on total RNA extracted from the samples used for SSH. Reverse transcription was performed using the superscript III enzyme (Invitrogen). Specific primers for Q-RT-PCR were edited using the Light Cycler Probe Design Software version 1.0 (Roche Diagnostics) [see additional file 1, for primers sequences]. The following Light Cycler run protocol was used: denaturation program (95°C, 10 min), amplification and quantification programs repeated 40 times (95°C for 15 s, 60°C for 5 s, 72°C for 16 s), melting curve program (60-95°C with a heating rate of 0.1°C per second and continuous fluorescence measurement), and a cooling step to 40°C. Amplification of single highly specific products was verified (melting curve analysis). For each reaction, the crossing point (CP) was determined using the "Fit Point Method" of the Light Cycler Software 3.3 (Roche Diagnostics). PCR reactions were performed in duplicate and the mean values of the CP were calculated.

For each candidate gene, the level of transcription of the target gene (Tg) was normalized using the transcription rate of the reference gene (Rg). The Rg used in the present study is the 28s ribosomal RNA from *Symbiodinium *spp. (GenBank: AJ830930). The transcription ratio (R) was calculated according to the formula:



### Complete Open Reading Frame characterisation of the validated candidates

RACE PCR experiments were performed to characterize the complete Open Reading Frame (ORF) of the validated genes. Total RNA extraction and polyA+ purification were conducted on non stressed nubbin tissues (described above). RACE PCR experiments were performed using the SMART™ RACE cDNA Amplification Kit (Clontech). The final PCR amplification for the 5' and 3' ends was conducted using the Advantage 2 PCR Enzyme System (Clontech). PCR products were cloned, sequenced, and the sequences analysed as previously described.

### Determination of the organism (host or symbiont) expressing the candidate genes

We developed cross PCR experiments performed on DNA extracted from the holobiont (host plus symbiont) and from pure cultured zooxanthellae to determine the organism (host or symbiont) expressing each candidate gene. Holobiont and zooxanthellae DNAs were extracted using DNAzol reagent (Invitrogen). Specific primers (see additional file 1) were designed for all the validated candidates and the zooxanthellae-specific primers ss5Z (5'-GCAGTTATAATTTATTTGATGGTCACTGCTAC-3') and ss3Z (5'-AGCACTGCGTCAGTCCGAATAATTCACCGG) identified in a previous study [57], were used in the present one. PCR reactions were performed using the Advantage 2 PCR Enzyme System (Clontech). PCR products were loaded on 1% agarose gels.

### Peptide and antibodies

BSA-coupled peptides (Genpep, France) were used to immunize New Zealand rabbits as previously described [84]. Peptide sequences used were CAVARKSAPTRKQVWI and CFTMKFSTTPEVTFEM for PdC-Lectin and Pdcyst-rich proteins, respectively. Sera of immunized rabbits were collected and tested for the presence of specific Igs three months after the initial injection using ELISA [85] with uncoupled peptides adsorbed onto Maxisorp plates (Nunc). IgG fraction was purified by affinity chromatography [86] and antibody specificity was tested by Western blot as previously described in [87].

### Immunolocalization procedures

Tissues from unstressed colonies *Pocillopora damicornis *were processed following a procedure previously described in [88]. Tissues embedded in Paraplast were cut into thin section (7 μm), mounted on silane-coated glass slides stored at 4°C in a dry atmosphere.

Immunolocalization on tissue sections was performed according to a protocol previously described in [88]. Briefly, deparaffinized sections were incubated for 1 h at room temperature in saturating medium (1% BSA, 0.2% teleostean gelatine, 0.05% Tween20 in 0.05 mol.L^-1 ^Phosphate-buffered saline [PBS] pH 7.4). The samples were then incubated overnight at 4°C in a moist chamber with the purified IgG at 20 μg.ml^-1 ^in the preceding buffer. The primary antibodies surplus was discarded by multiple rinsing in the saturating medium and samples incubated for 1 h at room temperature with biotinylated anti-rabbit antibodies (secondary antibodies) diluted 1:250 in the saturating medium. After incubation, slides were rinsed with PBS pH 7.4, and samples finally stained for 15 min with streptavidin AlexaFluor 568 1:50 diluted (Molecular probes) and 4',6-diamino-2-phenylindole, DAPI (Sigma, 2 μg.ml^-1^). Sections were mounted in Pro-Long antifade medium (Molecular Probes) and observed with a confocal laser-scanning microscope (Leica, TCS4D).

## Authors' contributions

JVD and GM conceived and coordinated the study, participated in molecular genetic studies (SSH, Q-RT-PCR) and wrote the manuscript. MA and DA participated in the design and the coordination of the study, drafted the manuscript. ER and DD participated in RACE-PCR experiments and DNA sequencing. LF and JVD helped design and conduct the experimental procedures in the aquaria. YM participated in the molecular modelling of PdC-Lectin. CFP participated in the measurements of zooxanthellae density and drafted the manuscript. ST, ET and DZ performed immunolocalization experiments and drafted the manuscript. All authors read and approved the final manuscript.

## Supplementary Material

Additional file 1 **Top blastx, annotation, GeneBank accession number and specific primers (Q-RT-PCR) of selected ESTs**. The data provided represents, Top blast, GeneBank accession number and specific primers used for Q-RT-PCR of selected ESTs. Click here for file
