# Supplementary material for: Coral bleaching under thermal stress: putative involvement of host/symbiont recognition mechanisms
Source: BMC Physiol. 2009 Aug 4;9:14. doi: 10.1186/1472-6793-9-14 (PMC2728513; doi:10.1186/1472-6793-9-14)
Supplement: Additional file 1 — Top blastx, annotation, GeneBank accession number and specific primers (Q-RT-PCR) of selected ESTs. The data provided represents, Top blast, GeneBank accession number and specific primers used for Q-RT-PCR of selected ESTs. [file 1472-6793-9-14-S1.doc]

| **Library/Cluster n°** | **Annotation** | **Top blastx :**  **Protein**  **[species]**  **(E-value)** | **GeneBank accession number** | **Putative functional class** | **Primer** |
| --- | --- | --- | --- | --- | --- |
| BRG cluster 12 | *Pdcyst-rich* | predicted protein [Nematostella vectensis]  (6e-04) | GH706859 | Cell/cell or cell/ligand interaction | F: ACATTCGAGATGAGGAACT  R: ATTGGAATCAGGAAACAGC |
| BRG cluster 26 | Putative *Flavodoxin reductase* | hypothetical protein [Photorhabdus luminescens]  (4e-04) | GH706874 | Photosynthesis | F: AGCATATTCATTTCGTGGT  R: CGCTTCGACAGGTCAA |
| BRG cluster 27 | *Mannose binding lectin-like* | mannose-binding lectin  [Acropora millepora]  (8e-22) | GH706875 | Cell/cell or cell/ligand interaction | F: ATTGGCAGAACGGAAG  R: GGGAGGAGACCTGGTA |
| BRG cluster 33 | Unchacterized apicomplexan-specific serine rich low complexity-like | hypothetical protein [Cryptosporidium hominis]  (8e-32) | GH706882 | Conserved protein domain | F: TTTGAGCTGACCCTCG  R: AGTCACCATCCAAGAGT |
| BRG cluster 36 | *Arabidopsis thaliana zinc finger protein-like* | predicted protein [Populus trichocarpa]  (2e-09) | GH706885 | Intracellular signalling pathway | F: ATGGTATGTATGGACGAGA  R: CCCAGTATTGTTCATTGGC |
| BRG cluster 39 | *Alexandrium fundyense chloroplast ferredoxin-like* | chloroplast ferredoxin [Alexandrium fundyense]  (3e-25) | GH706888 | Photosynthesis | F: GCCATAGTCGAACTGC  R: GGAAGGCTTGGAATTGC |
| BRG cluster 48 | *Symbiodinium sp Bacl-2* | Bacl-2  [Symbiodinium sp. C3]  (8e-57) | GH706898 | Oxidative detoxification | F: TGCGCTACAATCTCATCG  R: GCATCACATGGGGCAA |
| BRG cluster 51 | *Mitofusin-like* | predicted protein [Nematostella vectensis]  (2e-10) | GH706902 | Cytoskelton structuring | F: ACCTCTGAAGAGTCGC  R:AGAGCACGATTTGACATCTA |
| BRG cluster 52 | *Dipeptidyl-peptidase-like* | predicted protein [Nematostella vectensis]  (7e-39) | GH706903 | Protein degradation | F:TGTGATACCTCAACCAAACT  R: GGATGCTCTCAGATCAAG |
| BRG cluster 59 | *Alpha tubulin-like protein* | Tubulin alpha-1 chain [Lepeophtheirus salmonis]  (4e-04) | GH706910 | Cytoskelton structuring | F: GTTCTCTGAGGCTCGT  R: ACACAGCAGCATGAAT |
| BRG cluster 60 | *Putative CUB SUSHI domain* | PREDICTED: similar to intrinsic factor-B12 receptor  [Ciona intestinalis]  (8e-01) | GH706912 | Conserved protein domain | F: TGCAAATCGTTGCGCC  R: CCATACTGAACGTGACCA |
| BRG cluster 91 | *Ependymin-like* | hypothetical protein [Branchiostoma floridae]  (3e-07) | GH706946 | Cell/cell or cell/ligand interaction | F: CGTGACGGTTACTGAAA  R:AGCAAATCCATTAGCAATGAA |
| BIG cluster 6 | Putative calcium-binding protein | calmodulin III  [Euglena gracilis]  (1e-02) | GH706850 | Calcium Homeostasis | F: AGCATGGTGGACATGAA  R: CTCCATCGCTGTTGTGA |
| BIG cluster 7 | Aldo-keto reductase-like protein | aldo-keto oxidoreductase [Thalassiosira pseudonana]  (5e-22) | GH706853 | Oxidative detoxification | F: CAGCCTGATGTTTGGG  R: ATAGGAAAGGCGTGGT |
| BIG cluster 8 | *Karlodinium micrum* beta-tubulin-like protein | beta tubulin [Dinophyceae sp]  (5e-63) | GH706854 | Cytoskelton structure | F: TCCTTGCGAACGACAT  R: CTTTCACGATACCATTGCT |
| BIG cluster 9 | *Karlodinium micrum* major basic nuclear protein-like | major basic nuclear protein  [Karlodinium micrum]  (1e-05) | GH706855 | Intracellular signalling pathway | F: ACGGGAAATGCCTTG  R: CTGAACACGTTGGCTGA |
| BIG cluster 11 | *Symbiodinium sp* putative *carbonic anhydrase* | unknown protein  [Symbiodinium sp. clade C3]  (2e-62) | [GH706797](http://www.ncbi.nlm.nih.gov/nucest/221332764?ordinalpos=1&itool=EntrezSystem2.PEntrez.Sequence.Sequence_ResultsPanel.Sequence_RVDocSum) | Photosynthesis | F: AACCCTTCTCGACTGC  R: GGAGGCGACTACGTTG |
| BIG cluster 16 | HSP90-like protein | heat shock protein  [Toxoplasma gondii]  (1e-36) | GH706802 | Chaperone protein | F: ACGAGACACGTTCAGT  R: GATTCTGTTCTTGCCC |
| BIG cluster 17 | ferritin-like protein | ferritin heavy chain [Branchiostoma lanceolatum]  (1e-50) | GH706803 | Metabolism | F: GGAACTCGCCCTCAAG  R: AGGACGTATTGTGTGG |
| BIG cluster 19 | Nucleolar GTP-binding protein-like | predicted protein [Nematostella vectensis]  (1e-19) | GH706805 | Protein synthesis | F: CCCGATGAGCCGTAAA  R: TGTCAGCTTCACCTGC |
| BIG cluster 32 | Putative *Hypoxia inducible factor* | hypothetical protein [Branchiostoma floridae]  (3e-05) | GH706820 | Intracellular signalling pathway | F: TGGTTGACTGTTGCC  R: AGCAAGGAGGAAGCTAT |
| BIG cluster 33 | Coatomer-like protein | hypothetical protein [Trichoplax adhaerens]  (1e-16) | GH706821 | Cytoskelton structuring | F: AACAATAACACAACGCCT  R: CCATCAGTTACTGGTCACG |
| BIG cluster 35 | *Peridinium limbatum alphatubulin-like* | alpha tubulin [Peridinium limbatum]  (3e-92) | GH706823 | Cytoskelton structuring | F: AATCTCCTCCAGGAACC  R: TGAAGTGCGACCCTC |
| BIG cluster 44 | Polyadenylate-binding protein-like | RNA binding protein  [*Zea mays*]  (8e-08) | GH706833 | Intracellular signalling pathway | F: AAGTTTCTGCATAGCTCC  R: CAGGGTCTTGGTTGACA |
| BIG cluster 45 | Zinc finger protein-like | GK18522 gene product  [Drosophila willistoni]  (1e-01) | GH706834 | Intracellular signalling pathway | F: CAAGGAAAAGCCGAAGAC  R: GTGGCTCTATGAGTGGT |
| BIG cluster 48 | *Symbiodinium sp* Peridinin-chlorophyll a-binding protein-lik*e* | Peridinin-chlorophyll a-binding protein  [Symbiodinium sp]  (6e-16) | GH706837 | Photosynthesis | F: TTGATTCCAGCGTCCC  R: AGCAATGTCGCAACTT |
| BIG cluster 49 | *Putative thiol-disulfide oxido/reductase* | unnamed protein product [Ostreococcus tauri]  (9e-02) | GH706838 | Oxidative detoxification | F: TCCGCCAATGTTGGAA  R: TGCCTTGAGCTACCAG |
| BIG cluster 53 | *Variable lymphocyte receptor-like* | hypothetical protein  [Branchiostoma floridae]  (7e-31) | GH706843 | Cell/cell or cell/ligand interaction | F: CAGTCTCCAGACAGTG  R: GTTGTTCAGCGCCAAG |
| BIG cluster 61 | *Symbiodinium sp. actin-like protein* | actin [Symbiodinium sp. clade C]  (4e-73) | GH706852 | Cytoskelton structuring | F: CGAATATCGACATCGCAC  R: CTGCATCTGAGAGCAGT |
| Housekeeping gene | *28S Ribosomal RNA* |  |  |  | F: AAGTACCATGAGGGAAAGA  R: CGCATCCGCAAATGTG |
